# Clade Ib: a new emerging threat in the Mpox outbreak

**DOI:** 10.3389/fphar.2024.1504154

**Published:** 2024-12-19

**Authors:** Shriyansh Srivastava, Laxmi, Khyati Sharma, Sathvik Belagodu Sridhar, Sirajunisa Talath, Javedh Shareef, Rachana Mehta, Prakisini Satapathy, Ranjit Sah

**Affiliations:** ^1^ Department of Pharmacy, School of Medical and Allied Sciences, Galgotias University, Greater Noida, India; ^2^ Department of Pharmacology, Delhi Pharmaceutical Sciences and Research University (DPSRU), New Delhi, India; ^3^ RAK College of Pharmacy, RAK Medical and Health Sciences University, Ras Al Khaimah, United Arab Emirates; ^4^ Dr. Lal PathLabs Nepal, Kathmandu, Nepal; ^5^ Clinical Microbiology, RDC, Manav Rachna International Institute of Research and Studies, Faridabad, Haryana, India; ^6^ Center for Global Health Research, Saveetha Medical College and Hospital, Chennai, Tamil Nadu, India; ^7^ Department of Microbiology, Dr. D. Y. Patil Medical College, Hospital and Research Centre, Dr. D. Y. Patil Vidyapeeth, Pune, Maharashtra, India; ^8^ Department of Public Health Dentistry, Dr. D.Y. Patil Dental College and Hospital, Dr. D.Y. Patil Vidyapeeth, Pune, Maharashtra, India; ^9^ SR Sanjeevani Hospital, Siraha, Nepal

**Keywords:** clade Ib, emerging threat, transmission, surveillance, prevention, outbreaks

## Abstract

Monkeypox, a zoonotic virus in the *Orthopoxvirus genus*, has drawn global attention for its impact on public health. In the current Mpox outbreak, a novel clade, Ib, has emerged as a significant and potentially fatal threat. This review examines the dynamics of MPXV transmission, person-to-person spread, and infection mechanisms, highlighting key risk factors. We explore the clinical features of Mpox, focusing on symptomology, illness duration, and the distinguishing characteristics of clade Ib compared to other clades. A critical analysis addresses diagnostic techniques and emphasizes the need for robust surveillance, particularly for clade Ib detection. We review recent prevention and treatment strategies, including antiviral drugs and vaccines, with a focus on clade Ib containment. The conclusion underscores the urgency of global collaboration to prevent and prepare for emerging threats like clade Ib and identifies crucial research paths and knowledge gaps. This review offers a comprehensive overview of clade Ib, covering its emergence, genetic traits, epidemiological impact, transmission patterns, clinical features, the role of Artificial Intelligence (AI) in outbreak management, detection challenges, and implications for public health response.

## Graphical Abstract

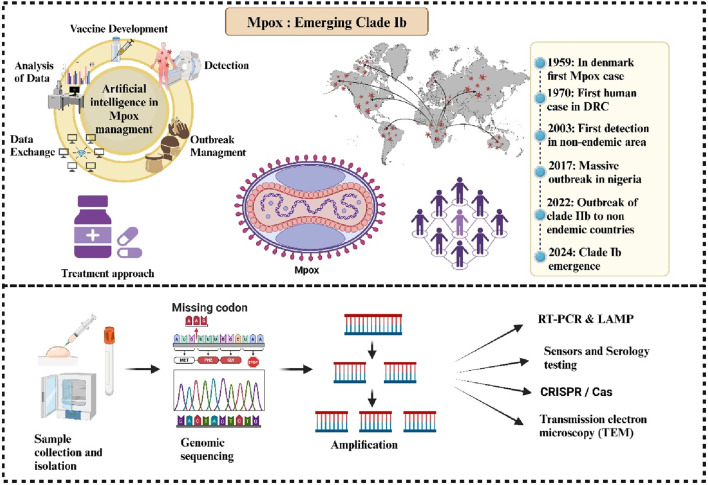

## Highlights

• Mpox clade Ib accelerates with contact and sexual transmission, putting children, immunocompromised people, and sex workers at risk.
• Due to genetic alterations, Mpox transmission has risen, requiring robust infrastructure for early identification and effective antiviral therapy.
• AI improves surveillance, diagnosis, and optimization for clade Ib outbreaks; data analysis identifies hotspots for greater precision and effectiveness.
• Lessons learned from COVID may reduce healthcare burden and stabilize economies, particularly in low-resource countries.

## 1 Introduction

Monkeypox, also referred to as Mpox, is becoming a more serious worldwide health issue, especially in a few African nations. The monkeypox virus (MPXV) was named for its clinical resemblance to smallpox, which was first observed in a Danish laboratory in 1958. The MPXV, the etiological agent, is a part of the *Orthopoxvirus genus* within the *Poxviridae* family and is native to West and Central Africa (Alakunle et al., 2020). MPXV is a brick-shaped, double-stranded DNA virus with a large genome that encodes most of its replication machinery. Recent studies have shown high viral loads in various bodily fluids, suggesting sexual transmission as a key driver of its spread (Moore et al., 2023). Clinically, Mpox symptoms present as rash, fever, and lymphadenopathy, potentially leading to complications such as bacterial infections, encephalitis, and pneumonitis (Srivastava et al., 2023). Close contact with infected humans or animals is the main way that the virus is transmitted, however, contaminated things can also spread the virus (Branda et al., 2024a). The significant rise in Mpox cases highlights the need for improved diagnostic procedures with high sensitivity, accuracy, and speed. Conventional and real-time PCR, serological tests, immunohistochemistry, TEM, and CRISPR-based technologies are effectively addressing this growing health issue (Zhou and Chen, 2023). Although antiviral therapy is approved for Mpox treatment, rapid case discovery, isolation of infected people, PPE use by healthcare personnel, providing vaccinations to risk populations and educating the public on hygiene and symptoms are crucial to preventing the Mpox from spreading (Kuehn et al., 2024). There are two main clades of MPXV: Clade I, discovered in the Congo Basin, has a higher virulence, while Clade II, discovered in West Africa, has a lower mortality risk (Happi et al., 2022; Martínez-Fernández et al., 2023). Clade IIb, comprising the lineages accountable for the epidemic in 2022, is now being overshadowed by the recently identified Clade Ib, which exhibits higher virulence and increased human transmission (Alakunle et al., 2024). Despite WHO ending the Mpox public health emergency in May 2023 (Lee et al., 2023), cases have surged in African nations, with over 3,600 confirmed cases and 32 deaths reported across 14 countries in 2024 (WHO, 2024a). Particularly vulnerable are Burundi, the Central African Republic, and the Democratic Republic of the Congo, where the newly discovered Clade Ib has contributed to rapid spread, exacerbated by factors such as mobility and humanitarian crises (Kozlov, 2024; Chavda et al., 2022). The effective management relies on rapid diagnostic advancements, targeted vaccinations, public education, and strict infection control practices, particularly in high-risk areas. Clade Ib enhanced human transmissibility and severity underscore the importance of global collaboration to contain outbreaks and address factors such as population mobility and regional crises that exacerbate the spread. Moving forward, coordinated research efforts and policy actions are essential to bridge knowledge gaps and strengthen resilience against future outbreaks.

## 2 Epidemiology of Mpox outbreaks

The genus *Orthopoxviruses* including the viruses that cause cowpox, vaccinia, variola, and Mpox. Among the African countries where Mpox is endemic are Benin, Cameroon, the Central African Republic, the Democratic Republic of the Congo, Gabon, Ivory Coast, Liberia, Nigeria, the Republic of the Congo, Sierra Leone, and South Sudan (Banuet-Martinez et al., 2023). In 1958, the MPXV was initially identified in the Copenhagen lab (Brasil et al., 2022). The first human instance of monkeypox virus infection involved a nine-month-old baby with smallpox symptoms, which was documented in the Democratic Republic of the Congo (DRC) in 1970. Between 1970 and 1990, there were over 400 instances of Mpox reported in Africa, with the majority occurring in the Democratic Republic of the Congo (Table 1). The DRC saw a protracted human monkeypox outbreak in 1996 (Heymann et al., 1998). Between February 1996 and February 1997, there were about 511 infected patients that underwent evaluation (Ullah et al., 2023). More than five hundred cases of monkeypox have been reported in 1999. Between 2001 and 2004, laboratories investigated 136 suspected Mpox cases, and 51 of them tested positive for the virus (Huang et al., 2022). In 2003, infected rodents accidentally spread monkeypox outside of Africa, where it was previously unknown, resulting in 34 lab-confirmed human cases and a total of 71 across six states. Genetic studies suggested the strain’s West African origin (clade II) contributed to its relatively low human-to-human transmission rate during the outbreak (Yu et al., 2023; Reed et al., 2004). Different numbers of cases of Mpox were documented in numerous African countries between 2010 and 2018. Nigeria was reportedly dealing with an outbreak in 2017. In 17 states in Nigeria, 122 cases of human monkeypox, either confirmed or suspected, were recorded between September 2017 and September 2018. There were six confirmed deaths (6% case fatality rate) (Kabuga and El Zowalaty, 2019). During the year 2018, Mpox made its initial excursion to the UK and was reported around Europe. There were two incidents reported involving people who had previously visited Nigeria (Mauldin et al., 2022). A public health emergency (PHEIC) was issued for Mpox on 23 July 2022, indicating a risk of international spread. Even though it’s still unclear what exactly caused the pandemic in 2022, the virus was probably imported from a nation where it was endemic, which would have allowed it to spread through close contact (Shafaati and Zandi, 2022). By March 2023, the 2022 Mpox outbreak had spread to 110 countries and 86,716 cases. Prior to the 2022 outbreak, the majority of areas, 103 out of 110 (94%) had no records of Mpox and after 2022, non-endemic areas, sickness and fatalities account for over 99% of cases. The US had the most occurrences overall, with about 30,000 and 20 deaths (CDC, 2023). America accounted for around 34.7% of all reported cases. Sexual interactions and household contacts (43%) caused the majority of infections. Young males who had sex with other men and had not received the smallpox vaccination were most affected (CDC, 2024e). The median age was 34 (IQR: 29–41), with a slight male bias; approximately 98% identified as bisexual, and 41% had HIV (Rimoin et al., 2010). The World Health Organization (WHO) keeps a careful eye on epidemics and responds by sharing information and cooperating with partners, and member countries, between January 2022 and November 2023, a total of 92,783 instances of Mpox and 171 fatalities were found and documented to the WHO from 116 nations, territories, and areas in 6 WHO regions (WHO, 2023b).

**TABLE 1:** Mpox Clades: This table compares geographical distribution, transmission modes, mortality rates, historical emergence, mutation rate, and public health impact for each clade, highlighting associated risk factors and global health implications.

Clades	Geographic distribution	History	Mortality	Mutation rate	Impact on public health	Transmission	Reference
Clade I a	Predominantly Central Africa (DRC)	Outbreaks in DRC and Central Africa	Highly virulent, with mortality rates of 1o-1o%	Stable genome, fewer mutations	Outbreak in endemic country and high fatality	Mostly zoonotic	WHO (2022)
Clade I b	Emerging variant in DRC	Newly identified with emerging outbreaks	Higher mortality than clade 2variant	Constantly mutating and evolving	Emerging, Declared a PHEIC in 2024	Household, contact, vertical, and heterosexual transmission	CDC (2024c) and WHO (2023a)
Clade II a	Mostly West Africa	outbreaks in Nigeria and other West African countrie	Lower virulence, <1% mortality	Moderately stable, with few variations	Infrequent outbreaks; lower fatality	Zoonotic and and restricted human to human transmission	NCDC (2021)
Clade II b	Global spread, also in non-endemic countries	Accountable for the 2022 global outbreak in America and Europe countries	Lowest virulence, <1% mortality in immunocompromised individual	Notably higher mutation rate, especially during global 2022 outbreak	Designated a Public Health Emergency of International Concern in 2022	Predominantly human to human transmission via lesions, infected objects and homosexual activities	ECDC (2022)

### 2.1 Clade Ib: characteristics and distribution

Since 2022, the number of Mpox instances and fatalities in the Democratic Republic of the Congo (DRC) has increased, over 100,000 confirmed laboratory instances and 220 fatalities have been documented in 120 countries across the world between January 2022 and August 2024 prompting the declaration of a Public Health Emergency of International Concern (PHEIC) two times, in May 2022 and August 2024. A concerning development has been the emergence of clade Ib, a novel variant of clade I, which spreads primarily through sexual networks and has been detected in neighbouring countries: Kenya, Rwanda, Uganda, and Burundi (CDC, 2024b; WHO, 2024b). In August 2024, over 100 confirmed instances of clade Ib were documented in these countries, which had not previously reported Mpox cases, with many more cases likely unreported (Post, 2024). This new strain first appeared among sex workers in Kamituga, DRC, in September 2023, and has since spread to nearby areas. Clade Ib is suspected to be more virulent and transmissible compared to other strains, which raises concerns about its potential global spread. August 2024 saw the appearance of the first verified instances of clade Ib outside of Africa in Stockholm, Sweden, and Thailand (Post, 2024; Branda et al., 2024b). This variant predominantly affects children through close household contact and has been linked to a higher number of deaths, with over 500 reported so far (Wenham and Eccleston-Turner, 2022; Fournier, 2018). Mutations in the virus, such as the APOBEC3 mutation, suggest a rapid evolutionary process, potentially increasing its transmission rate (Wenham and Eccleston-Turner, 2022).

## 3 Transmission dynamics

The possible methods of transmission are presently restricted to animal-to-person and person-to-person transfer. In central Africa, a variety of rodents and monkeys are the natural hosts of the Mpox virus. Initially, infections in humans are typically linked to interaction with animals that are infected, such as handling their enclosures, eating undercooked meat, or coming into contact with mucosal membranes, body fluids, or tissues. Additionally, scratches or bites from infected animals may spread the infection (Harris, 2022; O’Neill et al., 2023). MPXV may be transferred between humans via contact with lesions or monomorphic pustular rashes on the face, hands or genitals. It can be transmitted via the respiratory route (via droplets, talking or during breathing) as MPXV DNA was found in a PCR study of upper respiratory tract swabs from patients. Spread due to close contact or sexual transmission is also reported, contact of sores in the genitals, anus, and mouth, during sex may spread the virus (Kumar et al., 2022). MPXV DNA has been found in the semen and vaginal fluid of multiple patients, but it is still unclear whether it is a sexually transmitted disease or not (Garg et al., 2022). Before 2022, MPXV was mainly linked to clade II, especially haplogroup IIb, which showed lower severity and milder outcomes compared to clade I, and sexual transmission was insignificant. However, studies found that in non-endemic areas during the 2022 outbreak, sexual activities played a key role in spreading MPXV, particularly with the B.1 lineage of clade II adapting to transmit among humans, predominantly affecting men who have sex with men (Palich et al., 2023; Mailhe et al., 2023; Martínez et al., 2022). It can also spread vertically from infected mother to a child, during prepartum and *postpartum* period. Skin lesions were seen on body parts of new born babies and can also cause foetal death and miscarriages (Ramnarayan et al., 2022). Nosocomial transmission (e.g., needlestick injury during sample collection) has also been reported (Carvalho et al., 2022). Mpox Viruses can persist outside the body for extended durations, rendering surfaces like bed linens and doorknobs viable vectors for transmission (Milton, 2012). Unlike Clade II, which spread through sexual contacts, new emerging clade, clade Ib is said to transmit through close household contacts, and thus a disproportionately large percentage of children have been impacted in the Clade I outbreak, with over 500 documented fatalities to date (Ishoso et al., 2023). DRC findings indicate that heterosexual transmission, particularly among female sex workers, is driving the clade Ib outbreak. Due to its high prevalence in women, vertical transmission risks and pregnancy problems are significant concerns (CDC, 2024b). And so, this strain have again called for an emergency as Clade Ib is having higher transmissibility and potential for more severe clinical outcomes (Adepoju, 2024).

## 4 Clinical features

### 4.1 Symptoms and disease progression

**FIGURE 1:**
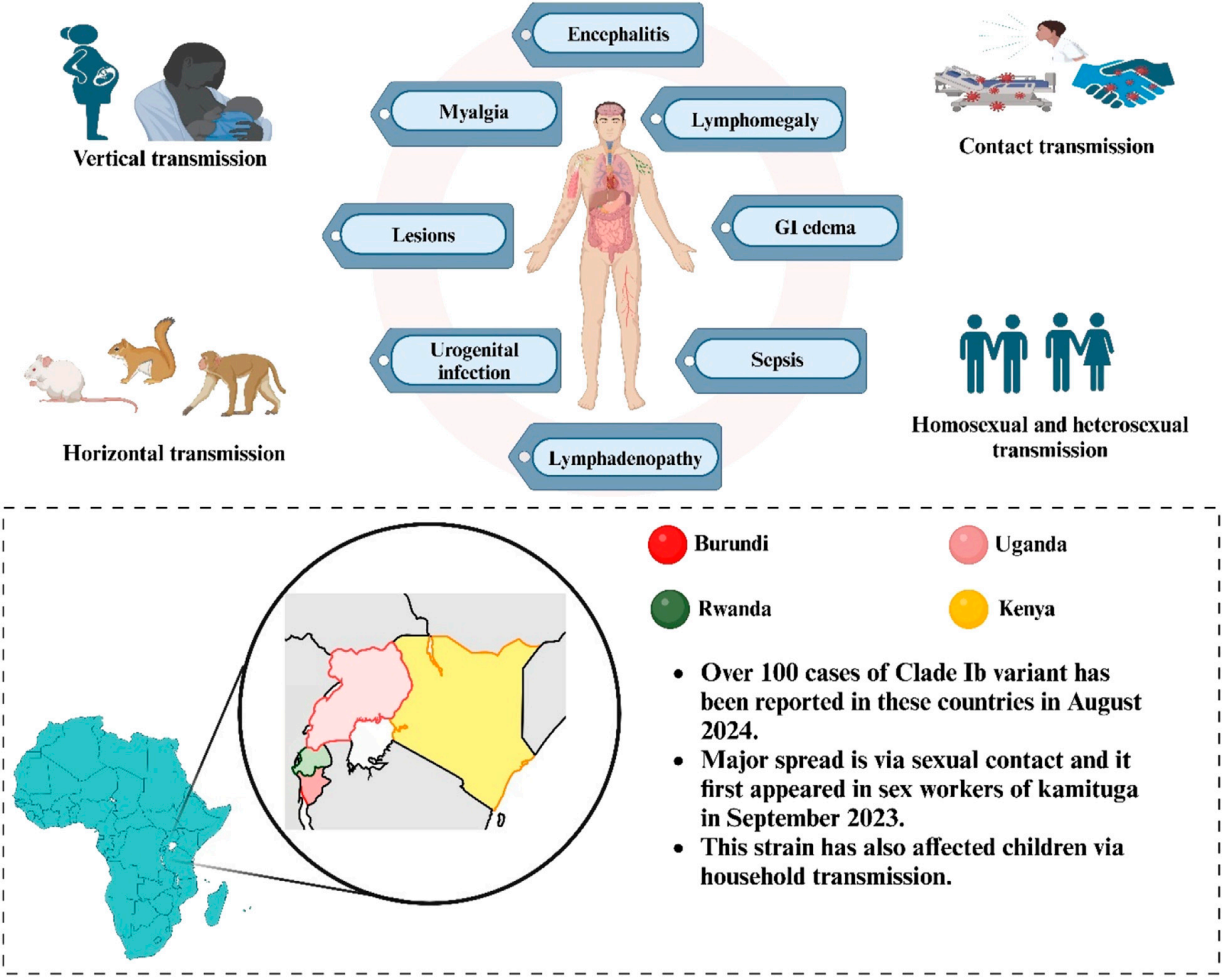
Routes of Mpox transmission (horizontal, vertical, contact, and sexual), stages of lesion formation, and clinical symptoms (encephalitis, myalgia, and lymphadenopathy). Highlights cases of Clade Ib reported in Burundi, Uganda, Rwanda, and Kenya.

The Mpox virus causes symptoms between 7–14 days, and the incubation period extend to 3 weeks (Ortiz-Saavedra et al., 2022). Symptoms include headache, fever, lymphadenopathy, malaise, coughing, throat irritation, rash, eye edema, oral ulcers, back discomfort, muscular soreness, joint discomfort, ocular and genital lesions, and oropharyngeal sores (Figure 1) (Mansoor et al., 2023). Following the incubation phase, an MPXV infection generally manifests with nonspecific systemic symptoms, such as fever, lasting approximately 2 days. This is typically succeeded by severe localized or localized lymphadenopathy 24–48 h before the emergence of skin lesions (Di Giulio and Eckburg, 2004; CDC, 2024a). One hallmark of the monkeypox virus that is thought to set it apart from other pox diseases is lymphadenopathy (Brown and Leggat, 2016). Generally speaking, lesions begin to show up 5–7 days after infection exposure. The skin lesions first manifest as an enanthem, or rash that advances via the macular, papular, vesicular, pustular, and scabious stages before desquamation. The lesions are deep-seated, rigid, and range in size from 2 to 10 mm. Lesions persist in the pustular stage for 1 week before crust development occurs. Crusts develop and shed between 1 week to 2 weeks, and in several instances, the infection resolves cures three to 4 weeks following the commencement of symptoms. The patient is considered to be no longer contagious until all of the crusts have fallen off (McCollum and Damon, 2014; Patel and Patel, 2023). Prior lesions were observed on the palms, soles, and face (Bunge et al., 2022). However, the traditional skin rash sites have changed during the Clade IIb outbreaks, with lesions or rashes also manifesting in the vaginal, perianal, and anal areas, as well as the trunk, upper limbs, oral, and peri-oral regions (Prasad et al., 2023). Moreover, studies also report lethargy and pharyngitis (Palich et al., 2023; Sharif et al., 2023). The monkeypox virus can cause bacterial superinfection (Mailhe et al., 2023), bilateral ocular issues, pneumonia (Vivancos-Gallego et al., 2022), parapharyngeal abscess (Girometti et al., 2022), oral ulcers, ARDS (Acute Respiratory Distress Syndrome), penile swelling sepsis, myocarditis, and conjunctivitis in infected individuals (Patel et al., 2023; Halwani, 2024). Skin infections and other secondary bacterial infections can lead to serious disease or fatality in persons with HIV/AIDS who have compromised immune systems. A “cytokine storm,” or a strong immunological reaction that harms the body, can be brought on by monkeypox (Johnston et al., 2015; Soheili et al., 2022).

### 4.2 Comparison of clade Ib with other clades

Divergences in virulence and pathogenicity among various clades arise from variations in their genetic composition and the evolutionary processes they undergo during interhuman transmission, alterations in ecosystems, interactions between humans and wildlife, and undetected circulation in wildlife across geographical territories. Additionally, more proficient viral strains may shape the evolving epidemiology of MPXV (Kumbhar and Agarwala, 2022). The virus has two clades, clade I and clade II, which include clades IIa and IIb, which transmit disease worldwide. Clade I was highly fatal and limited to Central Africa. In 2024, the new variant of clade I, clade Ib, is responsible for the MPOX pandemic. Some genes are absent or shortened in one or two clades, causing small gene content discrepancies. According to a recent study, Clade II contains three shorter genes (D4L, B14L, and B15L) and four missing genes (D14L, D15L, D16L, and D17L). The VACV-Cop E5R homologue is solely lacking in Clade I, and 3 of the genes (VACV-Cop A47L & B11R homologues and K1R) are truncated (Alakunle et al., 2024). In clade II APOBEC3 enzymes and some deleted genes can activate, adaptive development of Mpox towards increased human-to-human transmissibility. The Golgi-associated retrograde protein complex (GARP), N2R, and N3R genes were also linked to human-to-human transfer (Realegeno et al., 2017; Gao et al., 2023). The novel monkeypox virus (clade Ib) exhibits 54% more variation than clade I, with approximately 149 nucleotide changes. Cytosine deamination into uracil in viral genomes can occur, resulting in twice as many AT pairs as GC pairs, highlighting the role of APOBEC3 mutations in viral mutation and human transmission. (Table 1) (Martinez et al., 2019). A significant ∼1 kbp deletion in the MPXV genome, notably in the OPG032 gene, has been found in Kamituga epidemic genome sequences (Vakaniaki et al., 2024). Deleting this gene also prevents the US CDC’s Clade I-specific real-time PCR from detecting the virus (Li et al., 2010).

### 4.3 Vulnerable populations and associated risks

The Mpox virus can spread freely throughout all age groups and communities; however, it typically spreads to immunocompromised people, the elderly, children, and women faster. Furthermore, healthcare workers, like as physicians and nurses, are at a higher risk of transmission resulting from their regular interaction with sick patients (Masirika et al., 2024a). The Democratic Republic of the Congo is witnessing a significant incidence of illnesses among youth linked to clade Ib. Most fatalities and more than half of confirmed instances occur in children under the age of five, making children a vulnerable group for Mpox transmission. Epidemiologists have also identified transmission clusters associated with heterosexual or public sexual contacts (for example, sex workers in Kamituga), which were not popular during the clade II outbreak in 2022 (Katoto et al., 2024; Rivers et al., 2024). HIV-positive and immunocompromised people are especially vulnerable to infection. Individuals with advanced immune suppression from HIV may experience a serious disseminated form of Mpox characterized by necrotic skin lesions, pulmonary inflammation, secondary infections, sepsis, and prominent ocular manifestations such as periorbital illness (Mitjà et al., 2023; Vaughan et al., 2022). Non-infected Patients and healthcare workers are at peril from nosocomial MPXV infections. cases of transmission to healthcare worker have been reported from blanket and dressing of infected person (Vaughan et al., 2020). Laboratory workers are at significant risk of viral infection via unprotected exposure. However, adequate biosafety measures reduce the danger of infection, thus administrator or higher officials must undertake risk assessments at their facilities to determine safety precautions needed for Mpox (Adesola et al., 2023), pet raisers (Vaughan et al., 2020) and individuals who travel or participate in mass gatherings are at an even greater risk of infection, as the virus may be transmitted through touch or via airborne particles (Acharya et al., 2024). The LGBTQ+ community, including those who engage in homosexual behaviours (MSM), is more susceptible to spread infection (Amer et al., 2022). Therefore, it is imperative to guarantee that preventative strategies are effectively communicated to those who are most susceptible.

## 5 Impact of AI in managing the Mpox outbreak

**FIGURE 2:**
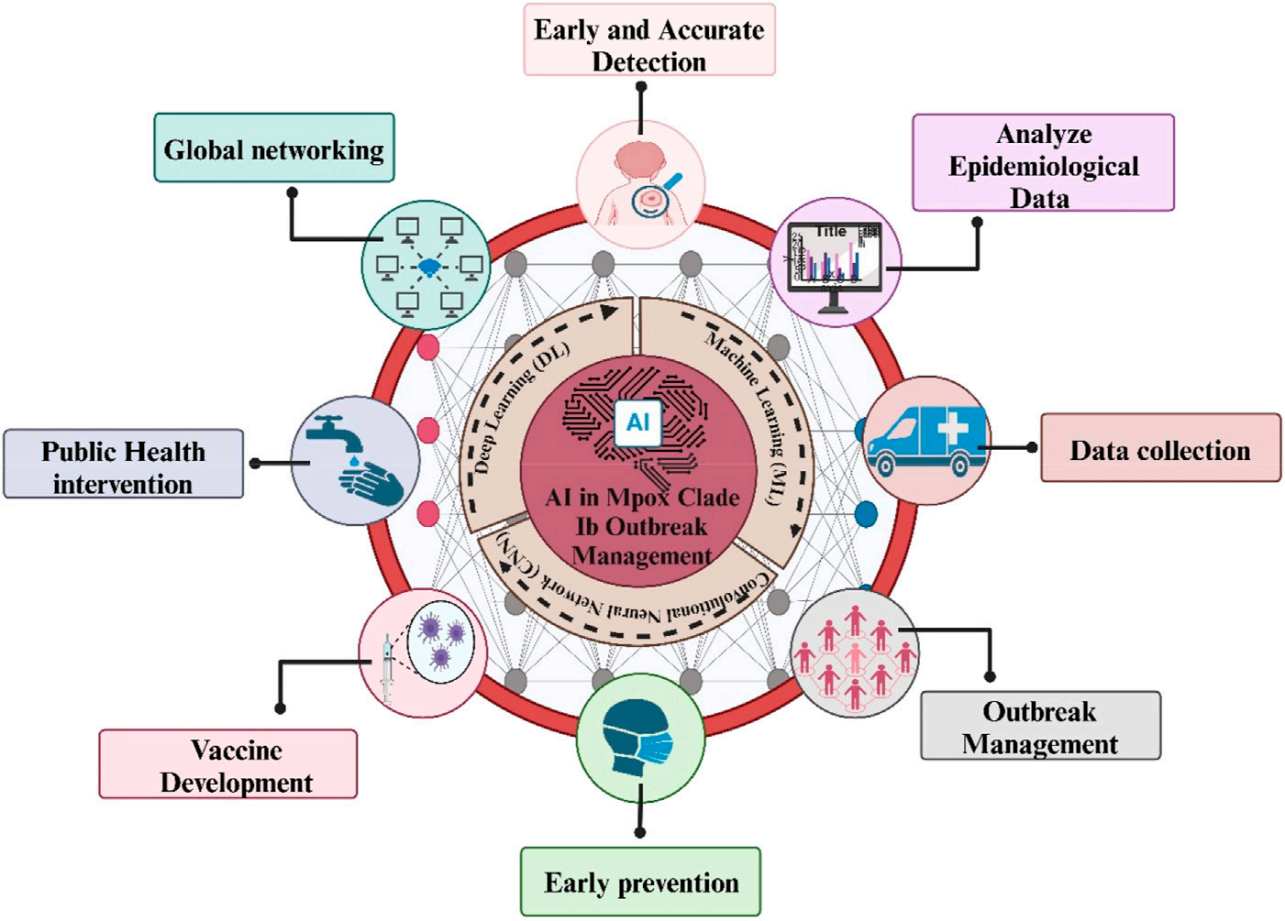
Artificial Intelligence (AI), including Machine Learning (ML), Deep Learning (DL), and Convolutional Neural Networks (CNN), manages Mpox Clade Ib epidemics by enabling early identification, analyzing epidemiological data, developing vaccines, and controlling outbreaks.

Recently, the WHO and Africa CDC deemed the outbreak to be a public health emergency of continental and global concern (Torales et al., 2024). Contrarily, clade Ib outbreaks appear to be primarily transmitted via sexual contact, with a substantial percentage of that interaction being heterosexual (Harris, 2024). Clade Ib instances have a risk of case fatality and manifest with a widespread full-body rash or persistent genital lesions (Rivers et al., 2024). There is a need to manage the Mpox outbreak. AI has improved several facets of the public health response, which has had a substantial influence on managing the Mpox clade Ib outbreak (Mendoza-Maldonado et al., 2024). AI-driven methodologies, deep learning (DL), and machine learning (ML), particularly when using convolutional neural networks (CNN), improve outbreak management, vaccination plans, and surveillance, especially for newly developing zoonotic diseases. Utilizing artificial intelligence (AI)-driven technologies, diagnostic primers are created for the quick and precise identification of Mpox, Clade Ib. These AI-created primers for the Clade 1b and the Mpox main lineage have been shown to be extremely sensitive, specific, and confirmed *in silico*. The ability to differentiate Mpox from other viruses depends on these primers (Mendoza-Maldonado et al., 2024; Nayak et al., 2023a). This allows medical practitioners to make prompt and well-informed judgments by enabling the automated analysis of medical imaging data to identify essential symptoms of Mpox, such as skin lesions and rashes (Nayak et al., 2023b). This capacity helps with early detection, which is essential for managing epidemics and halting further transmission, in addition to increasing diagnostic precision. Furthermore, epidemiological data may be analyzed by AI-driven models to forecast outbreak trends and guide public health interventions, which improves resource allocation and intervention tactics (Figure 2) All things considered, the use of AI in controlling the Mpox outbreak improves public health outcomes, helps quick reaction efforts and strengthens the efficiency of healthcare systems (Asif et al., 2024).

## 6 Diagnosis and surveillance

### 6.1 Diagnostic methods for Mpox

Many techniques have been employed in Mpox investigation to confirm the disease. These have been categorized into specialized methodologies for diagnosing Mpox in samples by PCR-based detection and serological techniques designed to identify virus-specific antibodies, virus isolation and culture, immunohistochemistry methods, Transmission electron microscopic, and CRISPR/Cas12b–gFET technique (Table 2) (Zhou and Chen, 2023).

**TABLE 2:** The multiple detection methodologies for the Mpox virus, including Real-Time PCR, serological methods, virus culture and isolation, transmission electron microscopy (TEM), CRISPR/Cas methodology, and CRISPR/Cas12b–gFET, highlighting their advantages and limitations for effective diagnostic assessment.

Detection techniques	Description	Advantages	Disadvantages	References
PCR and real-time PCR	Determine MPXV DNA from lesions and blood	Rapid, High sensitive, and specific determination of MPXV	Time-consuming, Requires specialized equipment	Jain et al. (2024)
Serological or antibody-detecting method	Detect IgG or IgM antibodies against Mpox virus in blood	Effective for assessing vaccine efficacy tests of epidemiological studies	Cannot differentiate between past and current, cross‐reactivity between *Orthopoxviruses*	Branda et al. (2024a)
Virus Culture and Isolation	Grow the Mpox in cell culture from the sample of patients	observable within approximately 24 h	Cytopathic effects	Branda et al. (2024a)
Immunohistochemistry (IHC)	Use antibodies to detect Mpox antigens in tissues	Provides detailed localization of viral antigens within tissues	Requires specific antibodies; time-consuming	Arunagiri et al. (2024)
Transmission Electron microscopy (TEM)	Visualizes viral particles in samples using electron microscopy	Less time consuming Large sample size	Requires high-level technical expertise and expensive equipment	Gentile and Gelderblom (2014)
CRISPR/Cas technique	Utilizes CRISPR/Cas systems to detect specific DNA	precise	Time-consuming	Yuan et al. (2021)
CRISPR/Cas12b–gFET	Combines CRISPR/Cas12b with graphene Field-Effect Transistor (gFET), used in the detection of the Mpox virus (MPXV) DNA	Rapid (about 20 min), Extremely high sensitivity	Requires specialized equipment for gFET; still in development stages	Wang et al. (2023)

#### 6.1.1 PCR and real-time PCR

According to the current guidelines from the WHO, the molecular detection of Mpox must rely on Nucleic acid amplification testing (NAAT) or genomic detection employing polymerase chain reaction or real-time PCR to identify distinct sequences of viral DNA in patient specimens (blood, lesions). Sequencing may be conducted either in combination with PCR or independently (Organization, 2022). The Mpox disease can be quickly, accurately, and specifically detected by utilizing oligonucleotide primers and a real-time PCR probe to detect viral genomes in sample material. For Mpox detection, different fluorescent probes used in real-time PCR assays. These probes have a quencher at the 3-prime end, such as minor groove binding (MGB), blackhole quencher 1 (BHQ1), or quencher succinimidyl ester (QSY7), and a covalently linked fluorescent-labeled reporter dye at the 5′end, such as tetrachloro fluorescein (TET) or 6-carboxyfluorescein (FAM) (Chauhan et al., 2023). Primarily, PCR is employed to validate the diagnosis of Mpox; however, alarmingly, recent data indicates that the US CDC’s approved Clade I-specific real-time PCR method may be affected by genetic alterations in the virus that is causing this outbreak (Bourner et al., 2024). Thus, the most reliable method for identifying this virus is PCR (Chadaga et al., 2023). The new Mpox Clade Ib was found by the validation and use of an innovative real-time PCR assay (dD14-16). The assay can be used to identify MPXV and differentiate between Clade I and Clade II in conjunction with other TaqMan-based real-time methods. For suspected instances of Mpox in Rwanda, a study evaluated the dD14-16 RT-PCR assay. It verified that Clade Ib was involved in every positive event in the generic CDC MPXV assay (Schuele et al., 2024).

#### 6.1.2 Serological or antibody-detecting methods

The application of serology, namely, identifying certain antibodies in plasma or serum, for the diagnosis of Mpox. Diagnosis was made utilizing complement fixation assays, neutralization assays, IHC, and/or ELISA-based diagnosis of the synthesis of Mpox-specific IgG or IgM antibodies in blood samples. Since these antibodies are indicators of acute immunological reactions, the IgM-detecting ELISA provides a means of detecting recent OPXV (Reynolds et al., 2010; Karem et al., 2005). In the context of OPXV species distinction, IgG ELISA is generally less effective, even though it helps detect vaccination efficiency testing and link epidemiologically characterized outbreaks (Chauhan et al., 2023; Yates et al., 2023). Serological cross-reactivity across *Orthopoxviruses* and The restricted supply of MPXV antigens and inactivated viral particles are the primary impediments to the advancement of Mpox serology (Kitamoto et al., 2005).

#### 6.1.3 Virus culture and isolation

This technique is essential for the diagnosis of viral illnesses, such as MPXV. Mammalian cell lines: Vero, BSC-1, RK-13, HeLa, along with chicken embryos, which are especially susceptible to poxviruses, exhibit robust proliferation for MPXV. One to 4 days after inoculation, the virus cause cytopathic reaction in the chorioallantois membranes (CAMs) of avian embryos. These effects include granulation, cellular rounding, cytoplasmic connectivity, and syncytium development. On the other hand, when grown in Vero cells, characteristic detached and rounded cells become visible in around 24 h, making it possible to identify viral particles using specific antibodies and immunofluorescence (Sah et al., 2024).

#### 6.1.4 Immunohistochemistry (IHC)

Immunohistochemistry remains the preferred technique for examining systemically Mpox infections in different human tissue samples, both currently and historically. MPXV antigens are identified by IHC using antibodies in biopsy and autopsy materials, while DNA-ISH is used to locate viral DNA in autopsy tissues. The distributions of antigen and DNA molecules were generally comparable, and the tissues and cells demonstrating significant localization to regions of tissue necrosis were also subjected to immunohistochemistry and *in situ* hybridization (Ritter et al., 2024).

#### 6.1.5 Transmission electron microscopy

It is a technique capable of confirming infection with MPXV from high-titer samples (vesicular fluids) in under half an hour, which is comparable to turnaround times using PCR techniques. Rapid Transmission Electron Microscopy is a Negative-contrast examination of vesicular material that can differentiate between species of the genus *Molluscum contagiosum* virus and the more frequently observed species of the genus *Parapoxvirus* or *Orthopoxvirus* (Lim et al., 2023).

#### 6.1.6 CRISPR/Cas12b–gFET technique

Using the Cas12 protein to precisely cleave MPXV DNA, the CRISPR/Cas technology is a promising molecular detection method. Because pre-amplification is required to target nucleic acids, CRISPR-based detection techniques necessitate lengthy test times (Lim et al., 2023; Zhao et al., 2024). A novel prototype Mpox biosensor that combines the CRISPR/Cas12b system with an ultrasensitive graphene field-effect transistor (gFET) to enable amplification-free nucleic acid detection. In roughly 20 min, the Mpox virus (MPXV) DNA targets could be found with a sensitivity of 1 a.m. thanks to CRISPR/Cas12b-gFET. Using a number of sgRNAs to identify the MPXV target genes, the Cas12b protein cleaved the genes to create a double-strand break (Wang et al., 2023; Mao et al., 2023).

### 6.2 Challenges in detecting clade Ib

The emergence of clade Ib, a new branch within clade I that is linked to both a high illness incidence and a high case fatality rate (CFR 4.9%–6.7%). Children under the age of 15, constituting about 70% of all recorded cases, are associated with around 88% of the fatalities in the current DRC outbreak. Clade Ib highlights the importance of ongoing observation and readiness because the virus’s dynamic nature may make identification difficult. There are genetic mutations in the genome of Clade Ib that appear to have been caused by the human immune system, indicating that the group has existed in humans for some time (Langat et al., 2024; Review, 2024). The mutational pattern found in the genome of new MPXV clade, clade Ib, indicates that cytosine deamination by the apolipoprotein B messenger RNA editing enzyme catalytic subunit 3 (APOBEC3) has been driving the virus’s evolution. Mutations associated to APOBEC3: a defining characteristic of person-to-person transmission of monkeypox virus (Vakaniaki et al., 2024). The following seven proteins were shown to be hotspots for mutations: C9L [OPG047], I4L [OPG080], L6R [OPG105], A17L [OPG143], A25R [OPG151], A28L [OPG153], and B21R [OPG210], demonstrating a range of unanimous in-frame modifications, frameshift variations, synonymous variations, and amino acid alterations (Masirika et al., 2024b). Certain MPXV gene loss events have been linked to person-to-person transmission, and these changes drive the virus' adaptive development toward increased transmissibility. The main technique for verifying Mpox, PCR testing, encounters challenges since these mutations may affect the specific RT-PCR tests that the US CDC recommends, decreasing their efficacy for Clade 1b. This is because not all of the assays that are currently available can be used efficiently, as evidenced by the fact that some primers, like G2R_WA, are not appropriate for this particular lineage. Furthermore, the robustness of the primer design may be impacted if a study relies too heavily on a small number of sequences (23 for Clade 1b) from the GISAID repository, which may not accurately reflect the genetic diversity of the clade (Mendoza-Maldonado et al., 2024). Furthermore, variations in the ecology of MPXV and the Golgi-associated retrograde protein (GARP) complex, as well as specific genes (N2R and N3R) and host cell signals and responses in West and Central Africa, were connected with the spread of the virus between individuals (Gao et al., 2023).

### 6.3 Importance of surveillance in outbreak management

The number of cases is significantly understated because of inadequate surveillance, improper routine identification, and other variables including asymptomatic infection, sexual transmission, and the significance of large gatherings (Tambo and Al-Nazawi, 2022). Conducting surveillance and quickly identifying new cases are essential to controlling an outbreak (Sukhdeo et al., 2022). Due to their tendency to spread worldwide, strengthening surveillance systems on a national and international level is crucial for early case identification and outbreak control. These cooperative initiatives are essential for exchanging information, pooling resources, and coordinating response plans among international partners and impacted nations (McQuiston et al., 2023). In order to manage Mpox epidemics, national and international cooperation is also necessary for the timely acquisition, distribution, and identification of any shortages in the supply of medical countermeasures (Zhang et al., 2023). It is crucial to carefully follow national and international regulations pertaining to the transfer of infectious materials while bringing samples to the lab. Individual samples must be meticulously prepared for transit using three layers of protection in compliance with WHO recommendations for the transportation of infectious materials (Patel et al., 2023). In order to enable quick data collecting and analysis, surveillance initiatives should incorporate syndromic surveillance, laboratory surveillance, and enhanced case-reporting procedures (Branda et al., 2022). Maintaining genomic surveillance activities is essential to identify and learn about the virus’s genetic alterations and promptly build preventative and control strategies (Yu et al., 2023). To successfully limit the effect of outbreaks, adequate preparation entails guaranteeing the accessibility of necessary medical supplies, such as PPE and antiviral drugs (Organization, 2023). To reduce the risk of transmission while providing patient care, healthcare personnel should be trained in the right use of PPE (Mutch et al., 2023). By raising knowledge, establishing trust, and promoting preventive measures in impacted areas, community involvement and risk communication are essential to reducing the impact of Mpox epidemics (Ugwu et al., 2022). Preserving a record of every person who has come into contact with humans or animals that have been confirmed to have Mpox is essential. Individuals should also be instructed to take their temperature two times a day and be watched for the onset of symptoms for 3 weeks after the last known exposure. Significant improvements in compliance processes can be achieved by providing personal supervision, instruction, checklists, feedback, and enough time for donning and doffing. These measures will help to greatly control the Mpox outbreak (Cices et al., 2023).

## 7 Treatment and prevention strategies

### 7.1 Antiviral therapies

Currently, the FDA has licensed two antiviral medications, Tecovirimat (TPOXX or ST-246) and Brincidofovir (CMX001 or Tembexa), as alternative treatment for those infected with Mpox. About 85% of people have been shown to be prevented against Mpox by smallpox immunization (Halwani, 2024; Gao et al., 2023). The US Food and Drug Administration (FDA) has authorized tecovirimat for the treatment of smallpox infections in children as well as adults. This drug blocks viral reproduction and release by impairing the correct function of VP37, a protein that wraps around viruses (Srivastava et al., 2023). Nowadays, tecovirimat’s efficacy in treating Mpox is being evaluated in a clinical phase called STOMP. Tecovirimat is advised for pregnant women who have contracted the MPXV, according to the Centers for Disease Control and Prevention (CDC). On the foetus, there are no particular negative effects (Isaacs et al., 2023). Tecovirimat-associated viral resistance has emerged due to mutations in the F13L gene. PAV-164, a replacement for methylene blue that can prevent the propagation of the Mpox virus, may be able to overcome this resistance (Zinnah et al., 2024). Brincidofovir is an prodrug of the cidofovir taken orally. Compared to cidofovir, brincidofovir might have a superior safety record.Beginning in June 2021, the FDA approved the brincidofovir for the treatment of smallpox (Hutson et al., 2021). It inhibits the DNA polymerase of virus, ultimately leading to the termination of the virus’s reproduction (Hudu et al., 2023). However, it was reported that after receiving 200 mg of brincidofovir orally once weekly for Mpox, three of the patients exhibited a rise in liver enzyme levels, which led to the treatment being discontinued (Patel et al., 2023).

### 7.2 Vaccination strategies and recommendations

Smallpox vaccination effectively provides cross-protection against MPXV and other poxviruses. Due to concerns about the MPXV outbreak and potential biological weapon attacks, authorities approved smallpox vaccine production in early 2021. Currently, the US Food and Drug Administration (FDA) has approved three vaccines: JYNNEOS (a non-replicating, live-attenuated vaccine, also known as Imvamune/Imvanex), ACAM 2000 (a replicating, live-attenuated vaccine), and LC16 (a slightly replicating vaccine) (Karagoz et al., 2023). In 2019, JYNNEOS (MVA-BN) received approval for use in the US and Canada following a number of animal studies (von Krempelhuber et al., 2010). When treating adults above the age of 18, JYNNEOS is given as two subcutaneous doses spaced 4 weeks apart to build immunity to prevent Mpox (Chakravarty et al., 2024). With an FDA expanded access use license for a single patient, pediatric children can utilize JYNNEOS. Crucially, in order to increase the available supply of vaccines, the FDA also approved the intradermal administration of JYNNEOS at a rate equivalent to one-fifth of the subcutaneous dose. One booster dose is necessary every 10 years for individuals exposed to low-virulence *Orthopoxviruses*, whereas those infected to highly virulent strains necessitate one every 2 years (Rao et al., 2022). Although the FDA approved ACAM 2000, the second smallpox vaccine, in 2007, it permits the virus to reproduce within cells. It has serious adverse effects as a result. Administered as a single percutaneous injection, ACAM2000 is a second-generation, live attenuated and replicable smallpox vaccine. Those with weakened immune systems, such as those living with HIV, its use is contraindicated and those receiving immunosuppressive or biologic treatments, pregnant women, individuals with previous episodes of cardiac disease because they are at risk of myocarditis and pericarditis, as well as those with a history of atopic dermatitis (AD) or other exfoliative skin conditions that impair barrier integrity. For this reason, it was purchased for the Strategic National Stockpile (SNS) and is now useable for a variety of demographic purposes (Cices et al., 2023; Decker et al., 2021). A license to use the LC16 (slightly replicating) vaccine, developed by KM Biologics, was obtained in 1975 for the prevention of smallpox in Japan and in 2014 for the elimination of Mpox in the U.S (Nishiyama et al., 2015). The highly immunogenic cellular protein B5R has been excised from this live-attenuated, slightly replicable vaccine, developed by cell-culture methods. The multidose vaccine is safe for individuals of every age group, including young children, is administered percutaneously using a bifurcated needle (Srivastava et al., 2023; Kennedy et al., 2011).

#### 7.2.1 Recommendation

• Updates on Mpox detection techniques and the preparation of appropriate PPE (gowns gloves, and face mask) for health workers, particularly in underprivileged areas (Safir et al., 2023).
• Healthcare professionals worldwide are keeping a close eye on foreign visitors who exhibit MPXV symptoms, particularly those involving fever, myalgia, and rash (European Centre for Disease Prevention and Control, 2022).
• To stop the spread of MPXV, disinfect affected surfaces with 0.1% of sodium hypochlorite (dilution 1:50) and launder garments and blankets at 60°C (Elkhwesky et al., 2023).
• To disrupt the transmission of MPXV, it is essential to understand the biological, social, and ecological connections between endemic and non-endemic (Gomez-Lucia, 2022).
• Designating sufficient financial resources to ascertain the mode of transmission of the disease, identify zoonotic hosts, determine MPXV, and analyze transmitter. The funding would make it easier to improve community readiness and provide first responders and medical workers with training (Ogunleye et al., 2024).
• Vaccination against smallpox in order to stop MPXV spread, as advised by the Centers for Disease Control (CDC) (Sah et al., 2022).

### 7.3 Public health measures for outbreak control

The viral disease Mpox poses a serious risk to public health, especially in nations with dense populations. Mpox does not have a specific treatment, but it can be controlled with supportive care, which includes antiviral drugs, antibiotics to stop secondary infections, and painkillers. In order to control outbreaks and lessen the effects of the illness, effective control measures are crucial (Islam et al., 2023). The identification and segregation of afflicted persons is the initial stage in the management of Mpox. Effective surveillance mechanisms should be set up by public health organizations to keep an eye out for outbreaks and make sure that any suspicious cases are looked into right away. Early detection and intervention can lessen the severity of the illness and stop its spread (Islam et al., 2023). The Centers for Disease Control and Prevention (CDC) advises medical professionals to take the appropriate precautions to lessen the spread of the virus and safeguard themselves. Standard PPE includes gowns, gloves, eye safety, and an NIOSH-approved particulate respirator with an N95 filter (Pediatric AAo, 2024). Patients with Mpox or exhibiting symptoms of infection should be kept apart and have their lesions covered in an outpatient setting. Patients who require hospitalization should wear masks; those who do not, especially young children and teenagers, should self-quarantine at their home. When performing procedures like intubation and extubation on patients that may cause them to produce oral secretions, medical professionals should employ a negative pressure chamber (Titanji et al., 2022). Providing a patient with an isolated bedroom and bathroom should be part of in-patient treatment. Individuals with the infection have to stay away from healthy people and animals until their scabs fall off, a new layer of skin grows, and the wounds are covered (Sanyaolu et al., 2023). The Mpox travel alert has been upgraded to Level 2 by the CDC, which advises travellers to take extra care because of outbreaks in both endemic and non-endemic regions. Travel operators and public health officials must work together to control risks, track down contacts, and advise travelers at entry points on how to recognize symptoms of Mumps, take preventative steps, and seek medical attention if necessary (Amer et al., 2023). Another crucial Mpox control method is vaccination. In certain epidemics, the smallpox vaccine, which guards against Mpox, has proven effective. Additionally, the CDC advises getting the JYNNEOS, ACAM 2000, or LC16m8 vaccine if they are 18 years old or older and at risk of direct exposure to Mpox. Antivirals for smallpox, such as tecovirimat and brincidofovir, have been made available by the Strategic National Stockpile because they may be useful in treating Mpox (Poland et al., 2022). Public health organizations should inform the public about the illness, how it spreads, and how crucial it is to get medical help right once if they think they may have come into contact with the virus. To stop the infection from spreading, people should be urged to maintain excellent hygiene, which includes often washing their hands (Rahman, 2024).

## 8 Impact on global health of Mpox outbreaks

### 8.1 Societal and economic implications of Mpox outbreaks

Mpox outbreaks have significant societal and economic implications, impacting public health, mental health, healthcare disparities, and social dynamics. The outbreak has intensified stigma, particularly against LGBTQIA+ individuals, due to the perception that it mainly affects men who have sex with other men (MSM). This stigma hampers public health communication and deters people from seeking testing or treatment due to fear of discrimination (Iglesias et al., 2022; Fan et al., 2024). Additionally, the outbreak has highlighted the influence of homophobia on public health narratives and the spread of misinformation and hate speech online (Fan et al., 2024). The psychological impact is notable, with increased fear and anxiety leading to heightened mental health issues. Effective mental health support strategies, such as telepsychiatry and stress management, are crucial (Torales et al., 2024). Economically, the outbreak has exacerbated healthcare access disparities, particularly affecting Black and Latinx communities, and imposed a financial burden on healthcare systems struggling with inequitable resource distribution (Plakkot et al., 2024; Chaudhari et al., 2023). The outbreak highlights the necessity for robust public health infrastructure and preparedness, including investments in surveillance, vaccine development, and equitable healthcare resource distribution. Inadequate preparedness can have substantial economic costs and affect societal stability (Torales et al., 2024; Chaudhari et al., 2023). Overall, the Mpox outbreak highlights the critical requirement for inclusive and equitable public health strategies to address stigma, ensure fair healthcare access, and support mental health, providing valuable lessons for future public health emergencies.

### 8.2 Leveraging COVID-19 insights to strengthen global Mpox response

The rising incidence of positive Mpox cases globally, amidst the ongoing COVID-19 pandemic, is a significant cause for concern. It is essential to discover and control Mpox virus transmission pathway in order to stop more illness outbreaks. Like COVID-19, MPXV is a health, political, and socioeconomic disaster that, if it is not contained quickly, will have detrimental effects on society. In the past 3 years, the fight against the COVID-19 pandemic has yielded invaluable experiences and learning that can be effectively implemented to prevent and control the re-emergence of the MPXV (Hemati et al., 2022). The COVID-19 pandemic made major progress in integrating genomic surveillance into public health procedures, which in turn led to the creation of the amplicon-based sequencing technique for the human monkeypox virus (Chen et al., 2023). The correlation between Mpox and COVID-19 is that during the pandemic, there was probably more awareness of and identification of Mpox cases due to heightened surveillance, which likely turned up instances that might not have been discovered otherwise, particularly in non-endemic areas. Furthermore, the simultaneous presence of Mpox and COVID-19 may exacerbate clinical presentations and public health responses, requiring cautious management and monitoring techniques (Yu et al., 2023). Similar to the Mpox and COVID-19 pandemic, public health is contingent upon the decisions made by government leaders, and individuals must persist in following health guidelines. Therefore, in order to stop global Mpox and measles outbreaks like COVID-19, governments and legislators need to implement the necessary preventative measures. The proficiency in disaster management is directly linked to the knowledge gained from ongoing experience, such as that from the COVID-19 pandemic and Mpox outbreak (Feitelson et al., 2022).

### 8.3 Strategies for future preparedness

Multiple steps must be taken to manage the Mpox outbreak, particularly for clade Ib. To conduct genomic surveillance of virus evolution and early diagnosis, it is imperative to improve surveillance and make molecular detection techniques more widely available. In order to lower the risk of spread, public health education is necessary for increasing knowledge about the symptoms, transmission dynamics, and preventative measures. Targeted vaccination programs, such as post-exposure immunization for contacts of confirmed cases and pre-exposure immunization regarding those at greater risk, can aid in halting transmission. High-risk populations and medical professionals must be the focus of treatment strategies that include antiviral medicine. The healthcare system with facilities for infrastructure and infection prevention tools like PPE (gloves, gowns, goggles, etc.). Timely national, and international cooperation are essential to address research deficiencies associated with Mpox epidemics. To finally understand the epidemiologic pattern of the monkeypox virus, international collaboration for improved surveillance and case identification is essential.

## 9 Research directions

### 9.1 Gaps in current knowledge about clade Ib

Clade Ib of the Mpox virus has become a major public health issue due to its transmission dynamics and evolving epidemiology. Despite ongoing research, critical knowledge gaps remain, hindering effective outbreak management. Uncertainty surrounds Clade Ib’s transmissibility, especially in non-endemic settings, with evidence suggesting sustained human-to-human transmission, particularly in urban areas. However, limited data exists on how its transmissibility compares to other clades, like Clade IIb, especially within high-contact sexual networks. The specifics of its incubation period, communicability, and disease severity in different populations also remain unclear (Go, Canada, 2024). Research on Clade Ib’s genetic diversity is limited, with early findings suggesting multiple introductions from animal hosts instead of a singular source. Understanding these genetic variations is essential for vaccine development and public health strategies (CDC, 2024d; Sciences Joi, 2024). The effectiveness of current public health interventions against Clade Ib remains uncertain. Although existing vaccines may offer some protection, their specific efficacy against Clade Ib needs further research. Additionally, improving public perceptions and vaccination uptake among at-risk populations is crucial for managing outbreaks (NIH, 2024). Finally, there are gaps in understanding the long-term impacts of Clade Ib outbreaks on health systems, mental health, and social structures. Addressing these gaps through focused research is critical for managing Clade Ib outbreaks and mitigating their global public health impact.

### 9.2 Future research priorities and funding opportunities

Future research priorities for the new Clade Ib should concentrate on several important areas including epidemiology, pathogenesis, transmission, pathways, and mutation patterns that are involved in Clade Ib, and the treatment of the disease. Since it can help in the development of specific vaccines and targeted treatments for Clade Ib. Furthermore, the development of reliable diagnostic tools specific for clade Ib should be the top focus of research. The development of lightweight models to enhance response time and lower computing costs will also be a focus of future studies. It is imperative to examine the socio-economic elements that are facilitating its transmission, especially in endemic areas. Funding opportunities for Mpox could be pursued by public health agencies, international organizations as well as national organizations, and private foundations with a focus on collaborative studies that integrate multidisciplinary approaches. Researchers can improve our understanding and control of Mpox clade Ib by collaborating with academic institutions, healthcare practitioners, and public health organizations. The European Union, the WHO, the CDC, the NIH, CEPI, and private foundations like the Bill and Melinda Gates Foundation are among the institutions that may provide funding for these study fields.

## 10 Conclusion

The emergence of Clade Ib of the MPXV calls for urgent global cooperation to combat the ongoing Mpox pandemic. Enhancing surveillance systems will improve detection capabilities, enabling faster and more effective outbreak responses. Targeted treatment and vaccination strategies, including antiviral drugs and vaccines specific to Clade Ib, are essential for managing this virus. Investment in research will fill critical gaps in understanding MPXV’s transmission dynamics, virulence, and mutation potential. Incorporating AI into public health strategies offers valuable support in data analysis, predictive modeling, and real-time decision-making, bolstering responses to emerging threats. Effective prevention strategies, including public health campaigns to educate communities on risk factors and promote vaccination, are crucial for reducing transmission. By addressing these challenges collectively, we can strengthen public health responses and build resilience against future zoonotic outbreaks. The global action is necessary to control Clade Ib of MPXV. Enhancing surveillance, developing targeted treatments, leveraging AI, and prioritizing prevention measures will help manage the current Mpox pandemic and prepare us for future challenges.

## Glossary

MPXV: Monkeypox virus
DNA: Deoxyribonucleic acid
PCR: Polymerase chain reaction.
TEM: Transmission Electron Microscopy
CRISPR: Clustered regularly interspaced short palindromic repeats
PPE: Personal protective equipment
WHO: World Health Organization
DRC: Democratic Republic of the Congo
PHEIC: Public Health Emergency of International Concern
IQR: Interquartile range
HIV: Human immunodeficiency virus
APOBEC3: Apolipoprotein B messenger RNA editing enzyme Catalytic subunit 3
ARDS: Acute Respiratory Distress Syndrome
GARP: Golgi-associated retrograde protein
CDC: Centers for Disease Control and Prevention
MSM: Men who have sex with men
AI: Artificial intelligence
DL: Deep learning
ML: Machine learning
CNN: Convolutional neural networks
CRISPR/Cas12b–gFET: Clustered regularly interspaced short palindromic repeats/Caspase12b-graphene field-effect transistor
NAAT: Nucleic acid amplification testing
MGB: Minor groove binding
BHQ1: Blackhole quencher 1
QSY7: Quencher succinimidyl ester
TET: Tetrachloro fluorescein
FAM: 6-carboxyfluorescein
ELISA: Enzyme-Linked Immunosorbent Assay
IgG: Immunoglobulin G
IgM: Immunoglobulin M
OPXV: Orthopoxvirus
CAMs: Chorioallantois membranes
IHC: Immunohistochemistry
sgRNAs: Single gRNA
CFR: Case-fatality ratio
RT-PCR: Real-time reverse transcriptase-polymerase chain reaction
GARP: Golgi-associated retrograde protein
FDA: Food and Drug Administration
VP37: Viral protein 37
STOMP: Study of Tecovirimat for Human Mpox Virus
MVA-BN: Modified vaccinia Ankara-Bavarian Nordic
AD: Atopic dermatitis
SNS: Strategic National Stockpile
NIOSH: National Institute for Occupational Safety and Health
COVID-19: Corona Virus Disease 2019
NIH: National Institutes of Health
CEPI: Coalition for Epidemic Preparedness Innovations
